# Factors associated with decision-making autonomy in healthcare utilization among married women from the Indonesia demographic health survey 2017

**DOI:** 10.1038/s41598-025-94057-3

**Published:** 2025-03-21

**Authors:** Sofa D. Alfian, Meliana Griselda, Mochammad A. A. Pratama, Sameer Alshehri, Rizky Abdulah

**Affiliations:** 1 Department of Pharmacology and Clinical Pharmacy, Faculty of Pharmacy, Universitas Padjadjaran, Jatinangor, Indonesia; 2 Center of Excellence for Pharmaceutical Care Innovation, Universitas Padjadjaran, Jatinangor, Indonesia; 3 Center for Health Technology Assessment, Universitas Padjadjaran, Jatinangor, Indonesia; 4 Department of Pharmaceutics and Industrial Pharmacy, College of Pharmacy, Taif University, Taif, Saudi Arabia

**Keywords:** Women, Autonomy, Healthcare, Decision-making, Demographic health survey, Indonesia, Diseases, Health care, Risk factors

## Abstract

Women’s autonomy in healthcare decision-making is crucial not only for improving maternal health but also enhancing their overall health and well-being. However, most studies focused solely on fertility, child health, or maternal healthcare use, often overlooking the broader aspects of women’s health. Due to this reason, the magnitudes and factors associated with women’s autonomy in other types of healthcare remain unclear. Therefore, this study aimed to estimate the magnitude and identify factors associated with healthcare decision-making autonomy among married women in Indonesia. A national cross-sectional study was conducted among married women using the Indonesia Demographic and Health Surveys 2017. Women’s healthcare decision-making autonomy was measured based on responses regarding the individual typically responsible for making healthcare decisions on behalf of the respondent. Potential factors, such as intrapersonal, interpersonal, community, and policy-related were obtained. Multinomial logistic regression was used to determine the associations between potential factors and outcomes. The odds ratio (OR) and 95% confidence intervals (CI) of the analysis were reported. The respondents in this study comprised 16,050 married women across 34 provinces in Indonesia. Most respondents (46.4%) reported making healthcare decisions independently. The result showed that several factors were associated with either women’s full autonomy or jointly with the husbands in the healthcare decision-making. These factors included ownership of mobile telephones, urban living, residency in Java, Bali, Sulawesi, Maluku and Papua islands, participation of women in decision-making on how to spend their earnings, on large household purchases, no financial barrier in accessing treatment, and independence in visiting a medical center. Public health interventions should focus on vulnerable women, such as those who live in rural areas, participate less in the decision-making of earnings spending and household purchase, and are incapable of visiting a medical center alone to increase the healthcare decision-making autonomy. Collaborative efforts with health facilities in each region can support the implementation of this intervention.

## Introduction

Women’s healthcare decision-making autonomy is very important in terms of both human rights and healthcare outcomes^1,2^. Autonomy in the context of healthcare decision-making is defined as the ability to act or make decisions without any limitations to the use of healthcare services and the choice among treatment options^3,4^. Healthcare decision-making autonomy has not only improved maternal health but also enhanced women’s overall health and well-being^3,5–8^. Prior study suggested that empowering women to make decisions about their healthcare can lead to improved mental health, especially in reducing anxiety^7^. Furthermore, another study revealed that autonomy in treatment decisions increases confidence in health management, resulting in sustainable and beneficial outcomes for patients^9^. Data from 57 countries showed that only 75% of married or partnered women aged 15 to 49 make their own healthcare decisions^10^. However, significant disparities exist across regions, with Eastern Asia, Southeast Asia, Latin America, and the Caribbean report higher percentages than the global average^10^, while developing regions such as Sub-Saharan Africa, Central Asia, and Southern Asia show comparatively low percentages of women making their own healthcare decisions^10^.

In developed countries, women with autonomy have a significant impact on maternal and child health^11^ by increasing healthcare visits and receiving appropriate treatment^3,5,6^. However, evidence suggests that women in developing countries tend to have limited autonomy in managing health decisions^11–14^ due to the rigid social structure that determines the roles of men and women^15,16^. In particular, the power inequalities at the household level between spouses are the most common types of social relationships that may restrict the health decision-making autonomy of married women^12^. Previous studies showed that restricting women’s autonomy in healthcare decision-making may lead to lower use of healthcare services in Ethiopia, Nigeria, and Ghana^1,17–19^. Furthermore, evidence from African countries showed that lower autonomy among women in healthcare decision-making is significantly associated with the risk of malnutrition^20^. In most Southeast Asian countries, women generally possess less power and autonomy than men in making decisions about healthcare^21^. Promoting the health and well-being of women is a critical issue, driven by the urgent need to address health disparities, particularly among internally displaced communities facing cycles of poverty and gender discrimination^22^. Traditional patriarchal culture significantly influences the decisions and actions of many women in Southeast Asia. A nationwide survey revealed that women with decision-making authority over their own healthcare were more likely to seek medical assistance than those whose spouses made such decisions^23^. Additionally, a study reported that Indonesian women with higher decision-making autonomy were more likely to attend antenatal care frequently compared to those with less power^24^^20^. Despite the importance of women’s healthcare decision-making autonomy, most studies in Indonesia focused only on fertility, children, or maternal healthcare use^25–27^. Consequently, the understanding of the extent and factors associated with the women’s autonomy in other types of healthcare use remain unclear^1^. This condition calls for attention as women tend to experience more morbidity than men^28−30^. Therefore, this study aimed to estimate the magnitude and identify factors associated with healthcare decision-making autonomy among married women in Indonesia.

## Materials and methods

### Study design and data sources

A community-based cross-sectional study was conducted using secondary data from the Indonesia Demographic Health Survey (IDHS) conducted in 2017. The data covers 34 provinces covering urban and rural areas in Indonesia obtained by Statistics Indonesia in collaboration with the National Population and Family Planning Board and the Ministry of Health of Indonesia^31^. Furthermore, this study included 47,963 households with an estimated number of 97,918 respondents and a high response rate of 94−100%^31^. The dataset is publicly available for free upon request from the DHS website (https://dhsprogram.com/)^32^.

### Study population and data collection

A two-stage stratified sampling was carried out with the first being the selection of census blocks by a systematic proportional to the size of households based on the 2010 census listing. Subsequently, the implicit stratification was carried out by sequencing census blocks based on urban and rural areas classified by the wealth index. In the second stage, 25 ordinary households were systematically selected in each census block^31^. Eligible respondents included women with several characteristics, such as (1) married aged 15−49 years, (2) living with the husband at the time of the interview, and (3) answered the question about who usually makes decisions on respondent’s healthcare. Women with incomplete data were excluded. The 2017 IDHS questionnaires were pre-tested before the fieldwork to test the validity and maintain the quality of data collected^31^.

### Conceptual framework

**Fig. 1:**
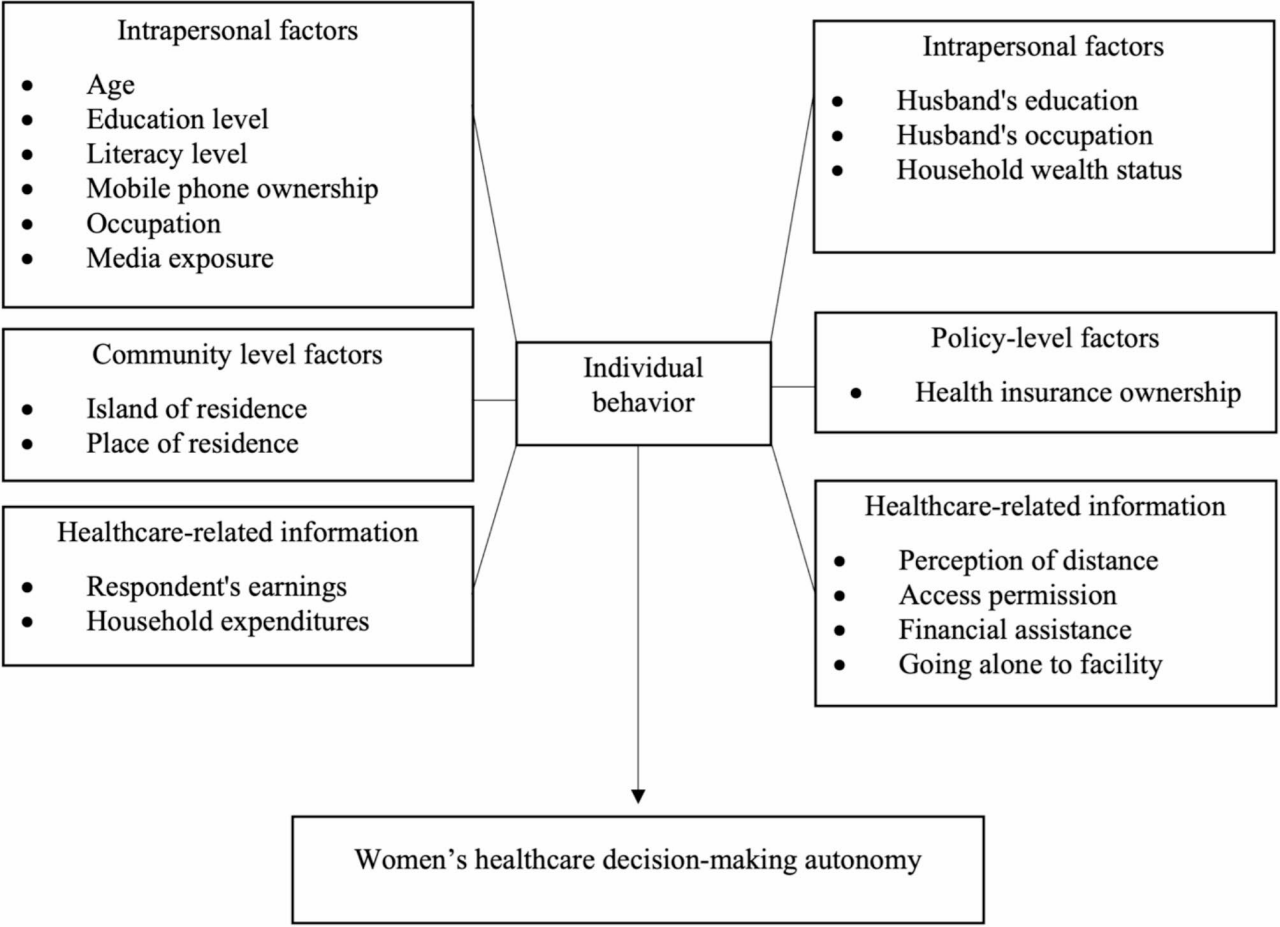
Conceptual Framework of Factors associated with Decision-making Autonomy in Healthcare Utilization among Married Women.

The conceptual framework was derived from existing literature on women’s decision-making autonomy^4,33–36^, as shown in Fig. 1.

### Women’s healthcare decision-making autonomy

The information on women’s healthcare decision-making autonomy was assessed using a self-report questionnaire with the question, “Who usually makes the decision regarding your healthcare?”. The response options include “respondent alone”, “respondent and husband”, or “husband alone”. Women who answered ‘husband alone’ were considered to have the least autonomy of the three groups. This group of women was contrasted with those whose response was “respondent alone” and “respondent and husband jointly”. This distinction is important because the dynamic process of autonomy represented by women reporting “sole decision-making” may differ from those reporting “joint decision-making with husband” or “by husband alone”.

### Potential factors associated with women’s healthcare decision-making autonomy

Potential factors associated with women’s healthcare decision-making autonomy were based on previous studies^4,33–36^. These factors included a total of 18 and were categorized according to the Socioecological Model^37^, which illustrated that individual behavior is influenced by factors at multiple levels: intrapersonal, interpersonal, community, and policy-related, as well as information related to healthcare decisions.

Intrapersonal factors represent individuals’ characteristics or individual-level factors^38^ and include the age of respondents (15–24 years, 25–34 years, or 35–49 years), education level, literacy level, ownership of a mobile telephone, occupation, and media exposure level. Interpersonal factors refer to the interactions between individuals that can influence behaviors^38^ and comprise the husband’s education level, husband’s occupation, and household wealth status. The household wealth status was determined based on the ownership of selected assets which was assigned a factor score from a principal components analysis for further standardization with mean and standard deviation of zero and one, respectively. The population quintiles of these standardized scores were then categorized into two categories, namely poor–middle, and rich^39^.

Community-level factors encompass the broader social and physical environment in which individuals live and interact^38^. These factors covered residency that was divided into two variables, namely island of residence and place of residence. The island of residence included Sumatera, Maluku and Papua, Java and Bali, Nusa Tenggara, Kalimantan, or Sulawesi. The survey database provided a comprehensive list of 34 provinces in Indonesia. However, due to low statistical power, we further categorized these provinces by grouping them into these major island clusters.

The place of residence categorized as either urban or rural. Furthermore, policy-level factors include laws, regulations, and organizational policies that influence health behavior at a larger scale^38^ and consist of ownership of a health insurance card.

Healthcare-related information was evaluated based on decisions pertaining to the allocation of the respondent’s earnings and significant household expenditures. Finally, medical help information for respondents was assessed by four factors, namely perception of distance to a health facility, access permission, financial assistance for treatment, and going alone to the health facility. The answer options for each item were “big problem” or “not a big problem”.

### Ethical approval and consent to participate

The Institutional Review Board of Inner-City Fund (ICF) International and ORC Macro (ICF IRB FWA00000845) granted ethical approval for the 2017 IDHS. Additionally, country-specific DHS survey protocols are reviewed by the ICF IRB and typically by an IRB in the host country. The ethical approval adhered to the U.S. Department of Health and Human Services requirement for the protection of human subjects^32^. Verbal informed consent was obtained from each respondent upon the interview and the given information was kept anonymous. The information about the ethical review is available on the DHS website https://dhsprogram.com/Methodology/Protecting-the-Privacy-of-DHS-Survey-Respondents.cfm (accessed on 8 November 2021).

### Data analysis

Given complex sampling design of the DHS survey, we applied complex sample analysis techniques for weighting prior to the analysis. This weighting statistically corrected for the unequal proportions identified during the sampling process. We conducted complete-case analyses to ensure the robustness and accuracy of our findings. Descriptive statistics were carried out to describe the socio-demographic information of the study population by using proportions and percentages. To assess multicollinearity, we calculated the variance inflation factors (VIF)^40^. Bivariate multinomial logistic regression was conducted to identify potential variables of women’s healthcare decision-making autonomy. The variables associated with the results at a significance level of *p* < 0.25 were then included in the initial multivariate model^41,42^. Multinomial logistic regression was carried out to obtain the odds ratio (OR) with a 95% confidence interval (95% CI). The significance threshold for a variable to be included in the final model was set at p-values of 0.05. Omnibus and Nagelkerke R-Square tests were applied to evaluate the final model. All statistical analyses were carried out using the Statistical Package for the Social Sciences (SPSS), version 27.0.

## Results

### Socio-demographic characteristics

A total of 16,050 out of 49,627 women who participated in the survey were married (Table 1). The majority were aged 35–49 years old (58.1%), had secondary or higher education (71.6%), classified as having poor to middle-wealth status (55.2%), were living in urban areas (57.0%), were covered by health insurance (66.2%), and were exposed to media at least once a week (57.1%) (Table 1). The majority of women reported insignificance concerning proximity to healthcare facilities (90.9%), encountered no obstacles in obtaining permission to access medical attention (95.7%), securing financial assistance for treatment (87.4%), and possessed the autonomy to access healthcare facilities unaided (80.2%) (Table 1).

**Table 1:** Respondent’s characteristics (*N* = 16,050).

No	Factors	*n* (%) Unweighted	% Weighted (95% CI)
1	Age (in years)		
	15−24	1240 (7.7)	8.3 (7.7–8.9)
	25−34	5482 (34.2)	33.2 (32.3–34.2)
	35−49	9328 (58.1)	58.5 (57.5–59.4)
2	Education level		
	No or primary education	4553 (28.4)	31.8 (30.5–33.1)
	Secondary or higher education	11,497 (71.6)	68.2 (66.9–69.5)
3	Literacy capability		
	Cannot read	1080 (6.7)	6.5 (5.9–7.1)
	Can read	14,970 (93.3)	93.5 (92.9–94.1)
4	Ownership of mobile telephone		
	No	3329 (20.7)	21.8 (20.7–22.9)
	Yes	12,721 (79.3)	78.2 (77.1–79.3)
5	Occupation		
	Not working/don’t know	22 (0.1)	0.1 (0.1–0.2)
	Clerk, service, and salesperson	9253 (57.7)	56.9 (55.6–58.3)
	Agriculture and industrial worker	4187 (26.1)	29.8 (28.4–31.3)
	Professional and manager	2588 (16.1)	13.2 (12.4–13.9)
6	Media exposure		
	Less than once a week	6883 (42.9)	46.7 (45.5–48.0)
	At least once a week	9167 (57.1)	53.3 (52.0-54.5)
7	Husband’s education level		
	No or primary education	4490 (28)	31.2 (29.9–32.5)
	Secondary or higher education	11,560 (72)	68.8 (67.5–70.1)
8	Husband’s occupation		
	Clerk, agriculture, not working/don’t know	14,080 (87.7)	89.5 (88.8–90.1)
	Professional and manager	1970 (12.3)	10.5 (9.9–11.2)
9	Wealth index		
	Poor and middle	8853 (55.2)	52.1 (50.5–53.6)
	Rich	7197 (44.8)	47.9 (46.4–49.5)
10	Covered by health insurance		
	No	5419 (33.8)	37.2 (35.9–38.4)
	Yes	10,631 (66.2)	62.8 (61.6–64.1)
11	Place of residence		
	Urban	9149 (57.0)	54.5 (53.2–55.7)
	Rural	6901 (43.0)	45.5 (44.3–46.8)
12	Island		
	Sumatera	4026 (25.1)	19.8 (18.9–20.8)
	Java and Bali	6075 (37.9)	62.7 (61.6–63.8)
	Nusa Tenggara	1515 (9.4)	6 (5.5–6.5)
	Kalimantan	1055 (6.6)	3.6 (3.3–3.8)
	Sulawesi	2221 (13.8)	5.9 (5.5–6.3)
	Maluku and Papua	1158 (7.2)	2 (1.8–2.3)
13	Person who usually decides how to spend respondent’s earnings		
	Respondent alone	11,508 (71.7)	73.2 (72.2–74.2)
	Respondent and husband	4031 (25.1)	23.8 (22.8–24.8)
	Husband alone	511 (3.2)	3 (2.7–3.4)
14	Person who usually decides on large household purchases		
	Respondent alone	2893 (18)	17.4 (16.6–18.2)
	Respondent and husband	10,101 (62.9)	62.3 (61.1–63.4)
	Husband alone	3056 (19)	20.4 (19.5–21.2)
15	Getting medical help for self: distance to a health facility		
	Big problem	1463 (9.1)	9 (8.3–9.8)
	Not a big problem	14,587 (90.9)	91 (90.2–91.7)
16	Getting medical help for self: getting permission to go		
	Big problem	697 (4.3)	4.5 (4.0-5.1)
	Not a big problem	15,353 (95.7)	95.5 (94.9–96.0)
17	Getting medical help for self: getting money needed for treatment		
	Big problem	2015 (12.6)	12.6 (11.9–13.5)
	Not a big problem	14,035 (87.4)	87.4 (86.5–88.1)
18	Getting medical help for self: going alone		
	Big problem	3178 (19.8)	20.6 (19.7–21.6)
	Not a big problem	12,872 (80.2)	79.4 (78.4–80.3)

### Healthcare decision-making autonomy of women

Regarding healthcare decision-making autonomy, 46.4% of women made decisions independently. Furthermore, 44.7% commonly made decisions jointly with the husband and only 8.9% depended on the husband for decision-making (Table 2).

**Table 2:** Results of bivariate and multivariate analysis.

No	Factors	Autonomy in healthcare decision–making							
		Bivariate				Multivariate			
		Respondent alone (*n* = 7,456; 46.4%)	Respondent and husband (*n* = 7,168; 44.7%	Husband alone (*n* = 1,426; 8.9%)	*p*-value	Respondent and husband AOR (95% CI)	*p*-value	Husband Alone AOR (95% CI)	*p*-value
1	Age (in years)				0.341				
	15−24	7.9 (7.1−8.7)	8.7 (7.8−9.6)	8.7 (7.1−10.6)					
	25−34	32.7 (31.4−33.9)	33.8 (32.4−35.1)	33.8 (30.9−36.8)					
	35−49	59.4 (58.1−60.8)	57.6 (56.1−59.1)	57.5 (54.4−60.6)					
2	Education level				0.000^a^				
	No or primary education	31.3 (29.7−32.9)	30.6 (29.0−32.4)	39.8 (36.3−43.4)		Ref		Ref	
	Secondary or higher education	68.7 (67.1−70.3)	69.4 (67.6−71.0)	60.2 (56.6−63.7)		1.07 (0.94−1.21)	0.285	0.90 (0.74−1.09)	0.269
3	Literacy capability				0.002 ^a^				
	Cannot read	6 (5.2−6.8)	6.4 (5.6−7.3)	9.3 (7.5−11.5)		Ref		Ref	
	Can read	94 (93.2−94.8)	93.6 (92.7−94.4)	90.7 (88.5−92.5)		0.98 (0.8−1.21)	0.855	0.94 (0.69−1.27)	0.684
4	Ownership of mobile telephone				0.000^a^				
	No	20.4 (19.0−21.8)	21.7 (20.3−23.2)	29.2 (26.2−32.4)		Ref		Ref	
	Yes	79.6 (78.2−81.0)	78.3 (76.8−79.7)	70.8 (67.6−73.8)		0.95 (0.84−1.08)	0.474	0.75 (0.62−0.92)	0.005^a^
5	Occupation				0.000^a^				
	Not working/don’t know	0.1 (0.0−0.2)	0.1 (0.1−0.3)	0.1 (0.0−0.7)		Ref		Ref	
	Clerk, service, and salesperson	59.1 (57.4−60.8)	54.2 (52.5−6.0)	58.1 (54.7−61.4)		0.62 (0.25−1.55)	0.308	0.65 (0.1−4.21)	0.648
	Agriculture and industrial worker	28 (26.4−29.7)	31.2 (29.4−33.0)	32.7 (29.4−36.2)		0.70 (0.28−1.75)	0.446	0.63 (0.1−4.07)	0.623
	Professional and manager	12.8 (11.8−13.8)	14.5 (13.4−15.6)	9.1 (7.6−11.0)		0.63 (0.25−1.59)	0.328	0.52 (0.08−3.46)	0.501
6	Media exposure				0.022^a^				
	Less than once a week	47.2 (45.5−48.8)	45.4 (43.7−47.1)	50.4 (47.0−53.9)		Ref		Ref	
	At least once a week	52.8 (51.2−54.5)	54.6 (52.9−56.3)	49.6 (46.1−53.0)		1.07 (0.97−1.19)	0.163	0.98 (0.83−1.15)	0.772
7	Husband’s education level				0.002^a^				
	No or primary education	30.7 (29.1−32.4)	30.5 (28.9−32.2)	36.6 (33.2−40.1)		Ref		Ref	
	Secondary or higher education	69.3 (67.6−70.9)	69.5 (67.8−71.1)	63.4 (59.9−66.8)		1.06 (0.94−1.19)	0.345	0.97 (0.80−1.17)	0.729
8	Husband’s occupation				0.231^a^				
	Clerk, agriculture, not working/don’t know	89.9 (88.9−90.8)	88.9 (87.9−89.8)	90 (88.0−91.7)		Ref		Ref	
	Professional and manager	10.1 (9.2−11.1)	11.1 (10.2−12.1)	10 (8.3−12.0)		1.10 (0.95−1.28)	0.184	1.17 (0.91−1.51)	0.218
9	Wealth index				0.000^a^				
	Poor and middle	49.7 (47.8−51.6)	53.5 (51.6−55.5)	57.5 (53.9−61.0)		Ref		Ref	
	Rich	50.3 (48.4−52.2)	46.5 (44.5−48.4)	42.5 (39.0−46.1)		0.90 (0.81−1.01)	0.067	0.95 (0.79−1.14)	0.569
10	Covered by health insurance				0.055^a^				
	No	36.4 (34.9−38.0)	37.2 (35.5−38.9)	40.7 (37.5−44.1)		Ref		Ref	
	Yes	63.6 (62.0−65.1)	62.8 (61.1−64.5)	59.3 (55.9−62.5)		0.96 (0.87−1.06)	0.416	0.97 (0.84−1.13)	0.736
11	Residence of living				0.000^a^				
	Urban	58.4 (56.6−60.1)	51.4 (49.6−53.3)	48.5 (44.6−52.5)		Ref		Ref	
	Rural	41.6 (39.9−43.4)	48.6 (46.7−50.4)	51.5 (47.5−55.4)		1.15 (1.02−1.30)	0.019^a^	1.23 (1.02−1.50)	0.035^a^
12	Island				0.000^a^				
	Sumatera	17.1 (16.0−18.2)	22.3 (20.9−23.7)	22.5 (19.7−25.6)		Ref		Ref	
	Java and Bali	66.5 (65.0−67.9)	58.7 (57.0−60.4)	61.8 (58.2−65.2)		0.72 (0.64−0.82)	0.000^a^	0.78 (0.64−0.96)	0.017^a^
	Kalimantan	5.3 (4.8−5.9)	6.7 (6.0−7.4)	6.4 (5.0−8.1)		0.86 (0.72−1.04)	0.120	0.88 (0.65−1.19)	0.403
	Nusa Tenggara	2.8 (2.4−3.1)	4.5 (4.0−5.1)	3.4 (2.6−4.4)		1.17 (0.94−1.46)	0.172	1.05 (0.75−1.45)	0.786
	Sulawesi	6.1 (5.6−6.6)	6 (5.4−6.5)	4.7 (3.8−5.7)		0.80 (0.68−0.94)	0.006^a^	0.76 (0.58−0.99)	0.043^a^
	Maluku and Papua	2.3 (1.8−2.9)	1.9 (1.7−2.2)	1.3 (0.9−1.8)		0.68 (0.49−0.94)	0.020^a^	0.41 (0.26−0.65)	0.000^a^
13	Person who usually decides how to spend respondent’s earnings				0.000^a^				
	Respondent alone	85.7 (84.7−86.6)	60.1 (58.5−61.7)	69.7 (66.7−72.5)		Ref		Ref	
	Respondent and husband	12.4 (11.5−13.3)	37.5 (35.9−39.2)	18.9 (16.5−21.5)		3.61 (3.24−4.02)	0.000^a^	1.76 (1.47−2.11)	0.000^a^
	Husband alone	2 (1.6−2.4)	2.4 (2.0−2.8)	11.5 (9.4−13.9)		1.93 (1.45−2.57)	0.000^a^	4.19 (3.04−5.77)	0.000^a^
14	Person who usually decides on large household purchases				0.000^a^				
	Respondent alone	27.8 (26.5−29.2)	6.4 (5.7−7.2)	14.3 (12.2−16.8)		Ref		Ref	
	Respondent and husband	51.5 (49.9−53.0)	81.3 (80.1−82.5)	30 (27.0−33.2)		5.82 (5.06−6.70)	0.000^a^	1.05 (0.84−1.30)	0.667
	Husband alone	20.7 (19.5−21.9)	12.2 (11.3−13.3)	55.7 (52.3−59.0)		2.16 (1.84−2.54)	0.000^a^	4.22 (3.40−5.24)	0.000^a^
15	Getting medical help for self: distance to a health facility				0.285				
	Big problem	9.1 (8.2−10.2)	8.6 (7.7−9.6)	10.4 (8.4−12.7)					
	Not a big problem	90.9 (89.8−91.8)	91.4 (90.4−92.3)	89.6 (87.3−91.6)					
16	Getting medical help for self: getting permission to go				0.072^a^				
	Big problem	4.3 (3.7−5.1)	4.3 (3.6−5.1)	6.2 (4.6−8.4)		Ref		Ref	
	Not a big problem	95.7 (94.9−96.3)	95.7 (94.9−96.4)	93.8 (91.6−95.4)		0.97 (0.74−1.25)	0.790	0.92 (0.61−1.39)	0.688
17	Getting medical help for self: getting money needed for treatment				0.183^a^				
	Big problem	13.1 (12.0−14.2)	12 (10.9−13.1)	13.4 (11.5−15.6)		Ref		Ref	
	Not a big problem	86.9 (85.8−88.0)	88 (86.9−89.1)	86.6 (84.4−88.5)		1.17 (1.01−1.35)	0.034^a^	1.32 (1.07−1.63)	0.010^a^
18	Getting medical help for self: going alone				0.000^a^				
	Big problem	17.5 (16.3−18.7)	22.4 (21.0−23.9)	28.8 (25.8−32.0)		Ref		Ref	
	Not a big problem	82.5 (81.3−83.7)	77.6 (76.1−79.0)	71.2 (68.0−74.2)		0.73 (0.64−0.83)	0.000^a^	0.59 (0.49−0.71)	0.000^a^

AOR = Adjusted Odds ratios; CI = Confidence interval.

^a^Significant factor (*p* < 0.05).

^b^Pseudo R-Square Test = 13.7%; Omnibus Test = *p* < 0.001.

### Factors associated with healthcare decision-making autonomy of women

All variables used in this analysis demonstrated no multicollinearity, with VIF values less than 10 (Table 3)^40^. The result of multivariate analysis showed that women who owned a mobile telephone were less likely to leave decision-making to the husbands (OR = 0.75; 95% CI = 0.62–0.92). Women who live in rural areas tend to include their husbands in healthcare decision-making (OR = 1.15; 95% CI = 1.02–1.30) or let their husbands make the decision alone (OR = 1.23; 95% CI = 1.02–1.50). Those who are domiciled in Java or Bali islands have a lower tendency to include their husbands in the decision-making process (OR = 0.72; 95% CI = 0.64–0.82) or let their husbands make the decision alone (OR = 0.78; 95% CI = 0.64–0.96). Furthermore, women who lived in Sulawesi, Maluku and Papua islands were less likely to make healthcare decisions jointly with their husbands (OR = 0.80; 95% CI = 0.68–0.94 and OR = 0.68; 95% CI = 0.49–0.94) or let their husbands make the decision alone (OR = 0.76; 95% CI = 0.58–0.99 and OR = 0.41; 95% CI = 0.26–0.65), respectively (Table 2).

**Table 3:** Variance inflation factors (VIF).

No	Factor	VIF
1	Education level	1.68
2	Literacy capability	1.28
3	Ownership of mobile telephone	1.30
4	Occupation	1.09
5	Media exposure	1.14
6	Husband’s education level	1.45
7	Husband’s occupation	1.12
8	Wealth index	1.39
9	Covered by health insurance	1.05
10	Place of residence	1.21
11	Island	1.06
12	Person who usually decides how to spend respondent’s earnings	1.03
13	Person who usually decides on large household purchases	1.03
14	Getting medical help for self: getting permission to go	1.12
15	Getting medical help for self: getting money needed for treatment	1.15
16	Getting medical help for self: going alone	1.06

Women who autonomously managed finances were also inclined to independently navigate healthcare decisions. According to Table 2, women collaborating with husband on financial matters tended to jointly make healthcare decisions (OR = 3.61; 95% CI = 3.24–4.02) or delegate choices solely to the husbands (OR = 1.76; 95% CI = 1.47–2.11). Additionally, those relinquishing full financial control to their husbands tend to yield complete control over healthcare decision-making to their husbands (OR = 4.19; 95% CI = 3.04–5.77) or jointly (OR = 1.93; 95% CI = 1.45–2.57). Women jointly overseeing significant household purchases tend to collaborate in healthcare decision-making (OR = 5.82; 95% CI = 5.06–6.70). Those deferring large purchase decisions tended to grant the husbands full authority over healthcare decisions (OR = 4.22; 95% CI = 3.40–5.24) or engage in joint decision-making (OR = 2.16; 95% CI = 1.84–2.54) (Table 2).

Women without financial barrier in accessing treatment were more likely to participate in joint decision-making (OR = 1.17; 95%CI = 1.01–1.35) and to entrust their healthcare decisions to their husbands (OR = 1.32; 95%CI = 1.07–1.63). Furthermore, this study showed that women perceiving independent access to healthcare facilities as feasible have a lower tendency to engage in joint healthcare decision-making (OR = 0.73; 95% CI = 0.64–0.83) compared to unilateral. These women were less likely to entrust healthcare decisions to the husbands (OR = 0.59; 95% CI = 0.49–0.71) than assuming full responsibility (Table 2).

The Omnibus test obtained *p* < 0.001, proving the viability and fulfillment of the multivariable analytic model. Moreover, the model’s components explained 13.7% of the women’s healthcare decision-making autonomy, according to the pseudo-R-Square test results (Table 2).

## Discussion

This study assessed the factors influencing women’s healthcare decision-making autonomy in Indonesia. Understanding women’s healthcare decision-making autonomy is crucial, as numerous studies have demonstrated its positive effects on women’s health and well-being. Conversely, a lack of autonomy is associated with poor mental health, decreased healthcare visits, malnutrition, and unmet family needs^8^.

The result of this study showed that most married women made healthcare decisions independently, followed by joint decision-making with their husbands and decisions made solely by the husband. The unilateral determination of decisions by the husband without the participation of the wife represents the lowest level of healthcare decision-making autonomy for women. In contrast, joint decision-making represents a level of partnership or equality. Joint decision-making is also an important aspect of decision-making processes for women, allowing husbands and wives to share consequences and respect preferences^43^.

The study showed that women who have mobile telephones were less likely to have healthcare decision-making by the husbands. This result is also consistent with previous studies conducted in Ethiopia^44^, Nigeria^2^, Sub-Saharan Africa^35^, and Pakistan^45^. Possession of a mobile phone could afford women greater exposure to media, potentially enhancing comprehension of healthcare information and the use of health facilities^35,44^. Furthermore, owning a mobile phone provides women not only with a wider access to information but also facilitates communication with family and friends, which can help enhance their self-confidence and bargaining skills within the household^46^. This empowerment enables women to actively participate in their healthcare decision-making^47^.

This study showed that women who actively participate in decision-making regarding the allocation of earnings tend to exhibit greater healthcare decision-making autonomy. Similar results were also observed regarding the large household purchasing decision-maker which showed significant association with healthcare decision-making autonomy. These results were consistent with the report of previous studies^48–51^. Improving the participation of women within households has led to enhanced decision-making regarding health insurance enrollment^52^ and maternal healthcare use^27,53^. Empowering women to participate in household decision-making can help boost their self-esteem and develop their bargaining skills^52,54^, thus enhancing their ability to make independent decisions regarding their healthcare. On the other side, the existing belief that husbands have the responsibility to make decisions in the household contribution led to less women’s healthcare decision-making autonomy^55^. Furthermore, men who predominantly manage the household finances create barriers for women in accessing medical care or transportation to healthcare facilities. This condition limits the ability of women to participate in healthcare decisions^12^.

The result of this study showed that lower healthcare decision-making autonomy of woman was associated with rural residency. This result is consistent with previous studies conducted in Ethiopia, Nigeria, and Bangladesh^2,5,56^. According to a previous study, patriarchy is often more implemented in rural regions, resulting in higher autonomy among males and may limit women’s access to education^57^. On the other hand, urban women are more educated and may have better access to the media and information^58,59^.

Women who lived in Java, Bali, Sulawesi, or Maluku and Papua islands were more likely to make healthcare decisions. This result is consistent with the report of a previous study on maternal health that women who lived in the provinces of Java and Bali had approximately two times higher probability of accessing adequate antenatal care and facility-based deliveries^27^. Public healthcare facilities and hospitals are more prevalent on the islands of Java, Bali, Sulawesi, Maluku, and Papua compared to other regions^60^. Moreover, the transportation systems on Bali and Java are well-developed and interlinked, whereas islands such as Sumatera and Nusa Tenggara continue to experience significant disparities^61^. These inequalities affect not only the tourism sector, resulting in diminished economic growth, but also hinder women’s mobility in accessing healthcare services^60^. This difference between islands might be due to the various contributions, particularly cultural differences. Early marriage continues to occur in certain islands in Indonesia, such as West Nusa Tenggara, Kalimantan, and South Sumatera, largely due to local traditional norms that promote matchmaking or forced marriages, especially among families from lower economic backgrounds, aiming to alleviate financial burdens^62^. However, early marriage is associated with a higher risk of school dropout, poorer economic outcomes, and limited women’s decision-making autonomy due to the inherent power imbalances within such marriages^7,62^.

Women who perceived financial access to treatment as “not a big problem” were more likely to engage in joint decision-making and to entrust their healthcare decisions to their husbands. The absence of financial barriers to accessing treatment may suggest that our respondents receive financial support from their husbands. As a result, their healthcare decision-making autonomy is likely to be either shared or entirely managed by their husbands^62^. Finally, women who perceived going alone to a health facility as “not a big problem” were more autonomous in healthcare decision-making. Previous studies also showed that the freedom of women to access healthcare facilities had a positive influence on healthcare decision-making autonomy^16,63,64^. Enabling women to freely access healthcare facilities will foster their receptiveness to health information and encourage direct discussions with healthcare professionals. This increased accessibility will help build their confidence and awareness regarding their health, ultimately enhancing their engagement in healthcare decision-making.

Based on the results, there is a need to target public health intervention to address intrapersonal factors, among married women to enhance healthcare decision-making autonomy. This intervention will lead to better healthcare outcomes since women gain more control over lives and health. Community-related factors should also be considered when designing and implementing the intervention. Additionally, policies that ensure safe and private access to health information and healthcare facilities for women is vital. Collaborative efforts with Community Health Centers in each region can help facilitate this intervention. Policy interventions aimed at enhancing women’s knowledge, behaviors, financial literacy, and resource management skills have shown positive results in strengthening their participation in household decision-making^65^. Furthermore, public health interventions that create supportive communities for women to share knowledge and express vulnerabilities have also demonstrated promising outcomes in empowering women to make independent decisions^8,66–68^.

To the best of our knowledge, this is the first study in Indonesia observing factors associated with women’s healthcare decisions-making autonomy that used nationally representative survey data with a massive sample size. Furthermore, there were differences in the distinction made between the dynamic process of autonomy represented by women reporting sole, joint decision-making with husband, and by husband alone. This distinction allowed for the identification of specific factors associated with separate processes of decision-making, which can be used to develop an intervention. Additionally, we applied complex sample analysis techniques for weighting the variables, which provided a statistical correction for the unequal proportions identified during the sampling process. However, this study faced some limitations, such as the restrictions of the cross-sectional nature on the inferences related to the causal relationship. The use of self-reported data may be subject to social desirability and recall bias. Furthermore, the unweighted results may limit the representativeness of the population and could potentially lead to under- or overestimations of the findings. Further studies using more advanced analysis, such as multilevel analysis and considering sampling weight are recommended to incorporate information regarding women’s autonomy in populations with different socio-economic and health-related characteristics, such as the number of living children, culture-related, type of disease(s), and behavior-related factors^12^. Qualitative studies are also needed to complement insights on social contexts, cultural practices, and other perceived barriers with respect to women’s autonomy.

## Conclusion

In conclusion, 44.7% of the respondents frequently collaborated with their husbands while 46.4% of them made independent decisions on their healthcare decision-making. Some factors identified in this study helped policymakers in improving women’s autonomy in the use of healthcare utilization. Public health interventions are needed to target less-participative women in financial decision-making and those who lived in rural to increase healthcare decision-making autonomy of women.

## Data Availability

The data used in this study are publicly available from the Indonesian Demographics and Health Survey (IDHS). The data can be accessed at https://dhsprogram.com/Countries/Country-Main.cfm? ctry_id=17.
